# Wnt/β-catenin signaling is a therapeutic target in anaplastic thyroid carcinoma

**DOI:** 10.1007/s12020-024-03887-0

**Published:** 2024-05-28

**Authors:** Diana Diaz, Kensey Bergdorf, Matthew A. Loberg, Courtney J. Phifer, George J. Xu, Quanhu Sheng, Sheau-Chiann Chen, Jamal M. Byrant, Megan L. Tigue, Heather Hartmann, Sarah L. Rohde, James L. Netterville, Naira Baregamian, Jeremy A. Goettel, Fei Ye, Ethan Lee, Vivian L. Weiss

**Affiliations:** 1 Department of Pathology, Microbiology, and Immunology, Vanderbilt University Medical Center, Nashville, TN USA; 2 Department of Pharmacology, Vanderbilt University, Nashville, TN USA; 3 Department of Biostatistics, Vanderbilt University Medical Center, Nashville, TN USA; 4 Department of Cell and Developmental Biology, Vanderbilt University, Nashville, TN USA; 5 Department of Surgery, Vanderbilt University Medical Center, Nashville, TN USA; 6 Department of Medicine, Vanderbilt University Medical Center, Nashville, TN USA

## Abstract

**Background:**

Anaplastic thyroid carcinoma (ATC) is a highly aggressive malignancy that has consistently shown Wnt/β-catenin (canonical) signaling activation in various study populations. There are currently no targetable treatments for *BRAF*-wildtype ATC and a lack of effective treatment for *BRAF*^V600E^ATC. Our aim is to identify whether Wnt inhibitors could be potential therapeutic agents for ATC patients with limited treatment options.

**Methods:**

In this Institutional Review Board-approved study, we utilize a cohort of 32 ATCs and 20 non-neoplastic multinodular goiters (MNG). We also use 4 ATC spheroid cell lines (THJ-16T, THJ-21T, THJ-29T, and THJ-11T) and two primary patient-derived ATC organoid cultures (VWL-T5 and VWL-T60). Finally, we use a murine xenograft mouse model of ATC for in vivo treatment studies.

**Results:**

Using a large patient cohort, we demonstrate that this near-universal Wnt signaling activation is associated with ligand expression- rather than being mutationally-driven. We show that pyrvinium pamoate, a potent Wnt inhibitor, exhibits in vitro efficacy against both ATC cell lines and primary patient-derived ATC organoids VWL-T5 (*p* < 0.05) and VWL-T60 (*p* < 0.01) Finally, using a murine xenograft model of ATC, we show that pyrvinium significantly delays the growth of ATC tumors in THJ-16T (*p* < 0.005) and THJ-21T (*p* < 0.001).

**Conclusions:**

We tested Wnt inhibitor treatment, both in vitro and in vivo, as a potential novel therapy for this highly lethal disease. Future large-scale studies utilizing multiple Wnt inhibitors will lay the foundation for the development of these novel therapies for patients with ATC.

Anaplastic thyroid carcinoma (ATC) is a rare but highly lethal form of thyroid cancer [1]. There is currently no targeted therapy for *BRAF*-wildtype ATC, and combination dabrafenib-trametinib therapy (approved for *BRAF*^V600E^ ATC) has shown limited efficacy to date [2, 3]. Lack of effective interventions has led to a dismal 5-year survival rate of only 5% and a median survival post-diagnosis of 4-6 months [4].

Interestingly, it has been shown in multiple study populations that ATCs consistently exhibit elevated Wnt/β-catenin signaling (hereafter referred to as Wnt signaling) [5–7]. Analysis of a large patient cohort from Vanderbilt University Medical Center (VUMC) confirms elevated Wnt signaling through both RNA sequencing analysis (32 ATCs) and nuclear β-catenin staining (Fig. 1a 16/17 ATCs, 94%, for immunohistochemistry (IHC)). Notably, despite this increased signaling, our recently published mutational analysis of this cohort detected only rare Wnt pathway-activating mutations (2/34 ATC samples, 6%) [8]. The lack of activating mutations, in combination with elevated Wnt signaling, led us to explore Wnt ligand expression. We identified significantly higher levels of seven Wnt ligands in ATC compared to benign multinodular goiters (MNG) (Fig. 1b).

**Fig. 1:**
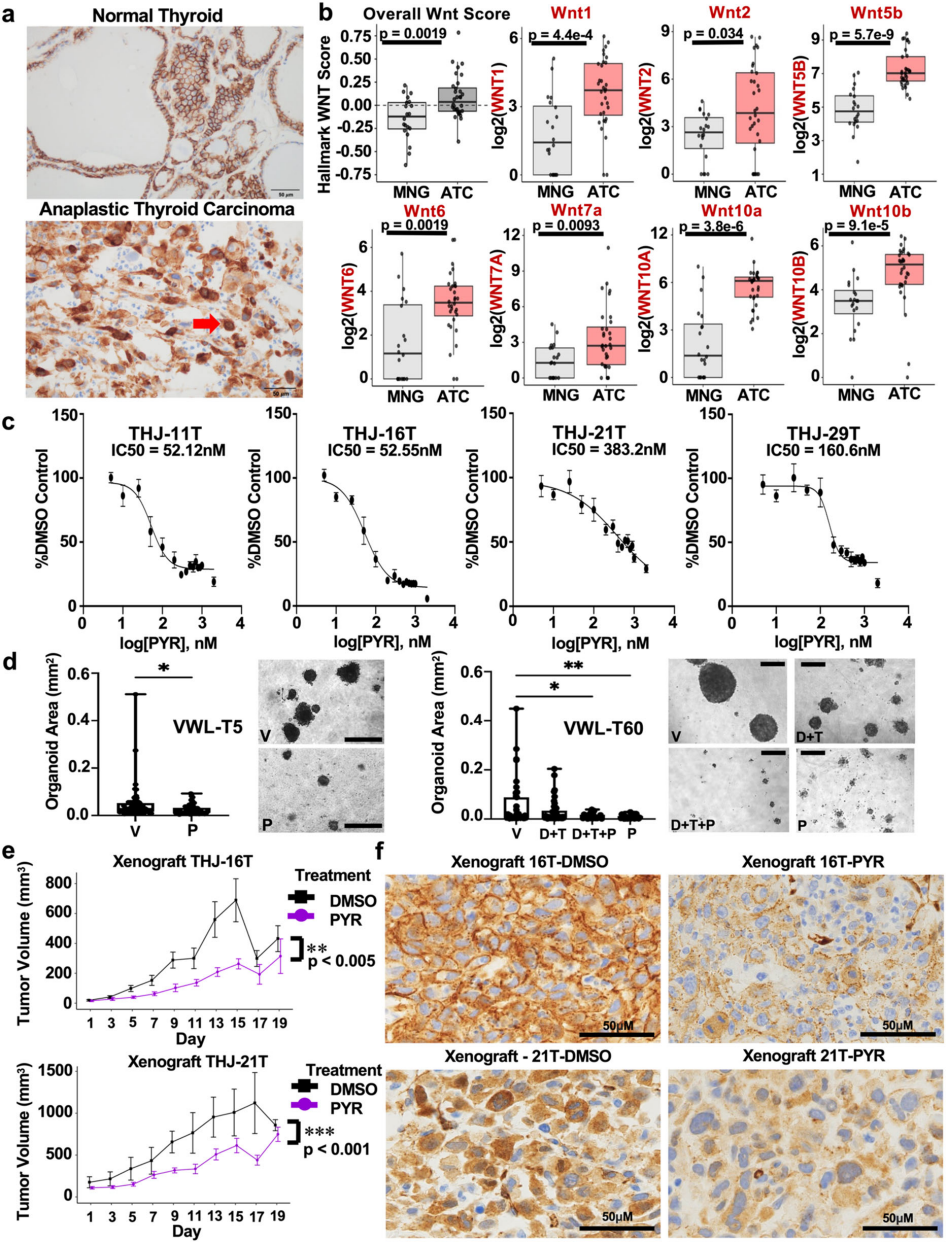
Targeted Wnt inhibitors as a potential therapy for Wnt-driven Anaplastic Thyroid Carcinoma (ATC). **a** β-catenin IHC staining in representative normal thyroid tissue (top) and ATC (bottom). The red arrow points to nuclear β-catenin staining. (scale bar = 50 µm). **b** Hallmark Wnt Score and Wnt ligand expression boxplots from RNA sequencing analysis of our patient cohort of 20 MNG samples and 32 ATC samples [8]. The significance for each plot was determined by the Wilcoxon rank sum test. **c** Normalized dose-response curves (±SEM) for spheroids treated with pyrvinium (PYR) for 72 hours (*n* = 3). IC50 is calculated by nonlinear regression. **d** Patient-derived organoid lines VWL-T5 (*BRAF*-wildtype) and VWL-T60 (*BRAF*-mutant) were treated with PYR for 8 days prior to organoid measurement. (V = vehicle, *P* = PYR, D + T = DAB + TRA, D + T + P = DAB + TRA + PYR; scale bar = 500 µm, * represents a *p*-value < 0.05, **p < 0.01) The significance of each plot was determined by unpaired t-test and one-way ANOVA. **e** NSG mice with subcutaneous flank tumors from xenograft THJ-16T (*n* = 52) or xenograft THJ-21T (*n* = 34). Tumor volume (±SD) was measured every other day. Both male and female mice were treated with PYR. Significance for both plots were determined by a mixed effect model. **f** β-catenin IHC staining of representative tumor tissue from vehicle-treated xenograft THJ-16T mice (top left), PYR treated xenograft THJ-16T mice (top right), xenograft THJ-21T vehicle-treated mice (bottom left) and PYR treated xenograft THJ-21T mice (bottom right) (scale bar = 50 µm)

Wnt signaling is classically associated with aggressive behavior across cancers. Increased Wnt signaling has been shown to support cell invasion, proliferation, and resistance to therapy. Given the lack of current effective therapy in this Wnt-driven disease, we aimed to determine whether targeted inhibition of Wnt signaling could be utilized as a potential therapy for ATC. Pyrvinium is an anti-pinworm drug that was identified as a potent Wnt inhibitor (CK1α agonist) [9]. It has been given orphan drug designation by the FDA for the Wnt-driven tumor syndrome Familial Adenomatous Polyposis and is currently in phase I clinical trial for pancreatic ductal carcinoma. Using a high-throughput ATC spheroid screen, we performed dose-response studies with pyrvinium (Fig. 1c) [10, 11]. Pyrvinium was more potent in xenograft THJ-11T and THJ-16T cell lines, in comparison to THJ-21T and THJ-29T.

To evaluate Wnt inhibition as a potential therapeutic approach, we utilized our fine-needle aspiration-derived primary patient tumor organoid cultures, which we have been shown to closely replicate patient tumor morphology and response to therapeutics [12–14]. As seen in the four ATC cell lines, pyrvinium significantly inhibited the growth of the *BRAF*-wildtype ATC organoid VWL-T5 (Fig. 1d). We also compared pyrvinium to current *BRAF*^V600E^ standard-of-care, dabrafenib + trametinib (DAB + TRA), in our VWL-T60 *BRAF*^V600E^ organoid culture (Fig. 1d). DAB + TRA combination therapy did not significantly decrease organoid size, consistent with clinical data demonstrating variable response to this therapy. In contrast, pyrvinium alone, and in combination with DAB + TRA significantly inhibited organoid growth. Finally, we used a murine xenograft model of ATC. Mice treated with pyrvinium showed decreased tumor growth and decreased nuclear β-catenin in tumor cells compared to mice receiving only vehicle (Fig. 1e, f). These data show the potential utility of Wnt inhibitor therapy as a novel treatment for ATC.

In defining the molecular drivers of ATC, we aim to identify druggable targets for patients who currently have few treatment options. In this study, we have delineated that increased Wnt signaling seen in our VUMC cohort of ATCs is not frequently mutationally driven, but commonly driven by increased Wnt ligands. Importantly, we demonstrate the efficacy of pyrvinium, a Wnt inhibitor, in spheroids, patient-derived organoid cultures, and murine models. These studies provide preclinical evidence for the effectiveness of Wnt inhibitors in the treatment of ATC, a lethal cancer with limited treatment options.

## Methods

### Patient cohort

A previously published cohort of thyroid resection specimens from Vanderbilt University Medical Center and the University Washington, including 32 ATCs and 20 MNGs, was used for RNA and histologic analysis [8].

### RNA sequencing

Computational analyses were performed with R version 4.3.1. Raw RNA count data from Xu et al was subset to ATC (32) and MNG (20) and normalized using DESeq2 1.40.2 [15]. A Hallmark Wnt signaling score was calculated as the average mRNA Z-score of the 42 genes in the hallmark Wnt signaling gene set from the Molecular Signatures Database (MSigDB) [16]. Hallmark Wnt scores and Log2 expression of Wnt ligands were compared between ATC and MNG using Wilcoxon rank sum tests and plotted using ggplot2 3.4.4 [17].

### Cell culture

Xenograft cell lines, THJ-11T, THJ-16T, THJ-21T, and THJ-29T, were obtained from Dr. John Copland (Mayo Clinic, Jacksonville, FL, USA) (refer to Supplemental Table 1 in Online Resource I). Cells were authenticated using STRS analysis and maintained and used experimentally at <20 passages from thaw. Cells were grown in complete media, which contains RPMI (VWR) containing 10% fetal bovine serum (FBS) (ThermoFisher Scientific), 1% penicillin-streptomycin (pen/strep) (Sigma), 1X MEM Non-Essential Amino Acids (VWR), and 1 mM sodium pyruvate (Vanderbilt Molecular Biology Resource).

### Generating dose-response curves

300 cells in 30 μL of suspension culture were plated per well in black 384-well cell-repellant culture plates (Greiner Bio-One). Spheroids were allowed to form for 24 hours prior to treatment with DMSO, dabrafenib+trametinib, and/or pyrvinium pamoate (Selleck Chemicals) and Matrigel in complete media. Following 72 hours of treatment, wells were imaged using an ImageXpress Micro XL automated high-content microscope (Molecular Devices). To assess viability, CellTiter-Glo 3D (Promega) was added to wells and mixed with the Bravo liquid handler (Velocity 11/Agilent). Per the CellTiter-Glo protocol, plates were placed on a shaker for 25 minutes before luminescence was quantified using a Synergy NEO (BioTek multi-mode plate reader) [11].

### Organoid culture

Patient-derived organoids were collected and maintained as previously described [12, 13]. For drug treatment, organoids were centrifuged for 5 minutes at 340xg to form a loose pellet. This pellet was washed with cold PBS prior to dissociation for 30 minutes at 37 °C in 1X TrypLE (Gibco). The resulting cell suspension were washed and resuspended in complete media containing 5% Matrigel and DMSO, 30 nM dabrafenib+5 nM trametinib, and/or 300 nM pyrvinium prior to plating in a 24-well low-attachment culture plate. Organoids were incubated at 37 °C, 5% CO_2_ for 8 days prior to imaging with a Leica DMi1 MC170 inverted microscope with a 4X objective and processed on Leica Application Suite, version 4.10.0. All work with patient-derived cells was approved by the Vanderbilt Institutional Review Board.

### Murine studies

All procedures were approved by the Institutional Animal Care and Use Committee prior to completion. NOD.PrkdcscidIl2rg-/- (NSG-Jackson Laboratories) were injected with 1 x 10^6^ xenograft THJ-16T or xenograft THJ-21T cells subcutaneously in the flank using a 25G SubQ needle affixed to a 1 mL syringe. When tumors became palpable (approximately 2 weeks post-injection), intraperitoneal injection of vehicle or pyrvinium pamoate (5% DMSO in corn oil) was started. To decrease the side effects of Wnt inhibition (colitis and fractures) and increase tolerability, we performed a dose-escalation of 0.5 mg/kg, 1 mg/kg, 1.5 mg/kg, 2 mg/kg with injections every other day, maintaining at 2 mg/kg for the remainder of the experiment. Injections were performed with a 25G SubQ needle on 1 mL syringes. Tumors were measured every other day using digital calipers, and mice were weighed weekly to ensure that weight loss did not exceed 20% of body weight. When tumors reached 2 cm in any dimension or ulcerated, mice were humanely euthanized. Tumor volumes were transformed to the square root scale to address skewness. A linear mixed-effects model was employed to account for the correlation inherent in repeated measurements within individual mice over time. Mouse ID was treated as random effect. The estimated growth rates from the model were compared between two treatments using Wald test. The analysis of tumor volume was conducted using R version 4.3.2.

### Generating stable cell lines

Stable Wnt reporter cell lines were generated using lentiviral transduction. Viral media was collected from HEK293FT cells transfected with the 7TFP lentiviral plasmid (Addgene #24308), along with the PAX2 (packaging) and pMD2G (envelope) plasmids. Thyroid cancer cell lines were cultured in lentiviral media with 8 mg/mL Polybrene for 24 hours. Antibiotic selection was performed with puromycin.

### TOPFLASH reporter assay

The TOPFLASH/7TFP reporter system was used to measure TCF/LEF transcriptional activity. Following Wnt treatment (16 hours), reporter cells were lysed in 1x Passive Lysis Buffer (Biotium) and incubated on a shaker for 15 minutes. Sample lysates were assayed for firefly luciferase reporter activity using Steady-Glo Luciferase Assay (Promega) and for cell viability using Cell Titer Glo (Promega). Luminescence was measured via FLUOstar Luminometer. Luciferase activity was normalized to cell viability. Assays were performed in triplicate and repeated independently at least three times (refer to Supplemental Figure I in Online Resource I).

## Supplementary information

Supplementary Information

## Data Availability

There are restrictions to the availability of patient clinical and sequencing data. This is a retrospective cohort, and it is not possible to consent these patients with historic samples, particularly those with highly aggressive and rapidly lethal disease. As such, the IRB has requested that we do not publicly share individual-level sequencing data from each patient. The data is securely stored within a Vanderbilt patient data system. Aggregate-level data reported in this paper will be shared by the lead contact (Dr. Vivian Weiss) upon request. Individual-level data is only available through collaboration following approval of the lead contact and The Vanderbilt University Medical Center IRB. If the lead contact should leave the institution, collaboration requests should be directed to the Pathology Department Chair (Dr. Alice Coogan, alice.coogan@vumc.org).
