# Supplementary material for: Wnt/β-catenin signaling is a therapeutic target in anaplastic thyroid carcinoma
Source: Endocrine. 2024 May 28;86(1):114–8. doi: 10.1007/s12020-024-03887-0 (PMC11444896; doi:10.1007/s12020-024-03887-0)
Supplement: Supplementary file 1 — Supplementary Information [file 12020_2024_3887_MOESM1_ESM.docx]

Wnt/β-catenin Signaling is a Therapeutic Target in Anaplastic Thyroid Carcinoma

Diana Diaz^1^*, Kensey Bergdorf^2^*, Matthew A. Loberg^1^, Courtney J. Phifer^1^, George J. Xu^1^, Quanhu Sheng^3^, Sheau-Chiann Chen^3^, Jamal M. Byrant^2^, Megan L. Tigue^1^, Heather Hartmann^1^, Sarah L. Rohde^4^, James L. Netterville^4^, Naira Baregamian^4^, Jeremy A. Goettel^1,5^, Fei Ye^3^, Ethan Lee^2,6^, Vivian L. Weiss^1,2^

1. Department of Pathology, Microbiology, and Immunology, Vanderbilt University Medical Center, Nashville, TN, USA

2. Department of Pharmacology, Vanderbilt University, Nashville, TN, USA.

3. Department of Biostatistics, Vanderbilt University Medical Center, Nashville, TN, USA

4. Department of Surgery, Vanderbilt University Medical Center, Nashville, TN, USA

5. Department of Medicine, Vanderbilt University Medical Center, Nashville, TN, USA

6. Department of Cell and Developmental Biology, Vanderbilt University, Nashville, TN, USA

* These authors contributed equally to this work

Corresponding author: vivian.l.weiss@vumc.org

**Supplemental Figure I**: Wnt reporter activation at different doses of Wnt3a in xenograft ATC cell lines: THJ-11T, THJ-16T, THJ-21T and THJ-29T. Significance for each figure was determined by a one-way ANOVA (* represents a p-value<0.05, ** p<0.01, *** p<0.001, and **** p<0.0001).


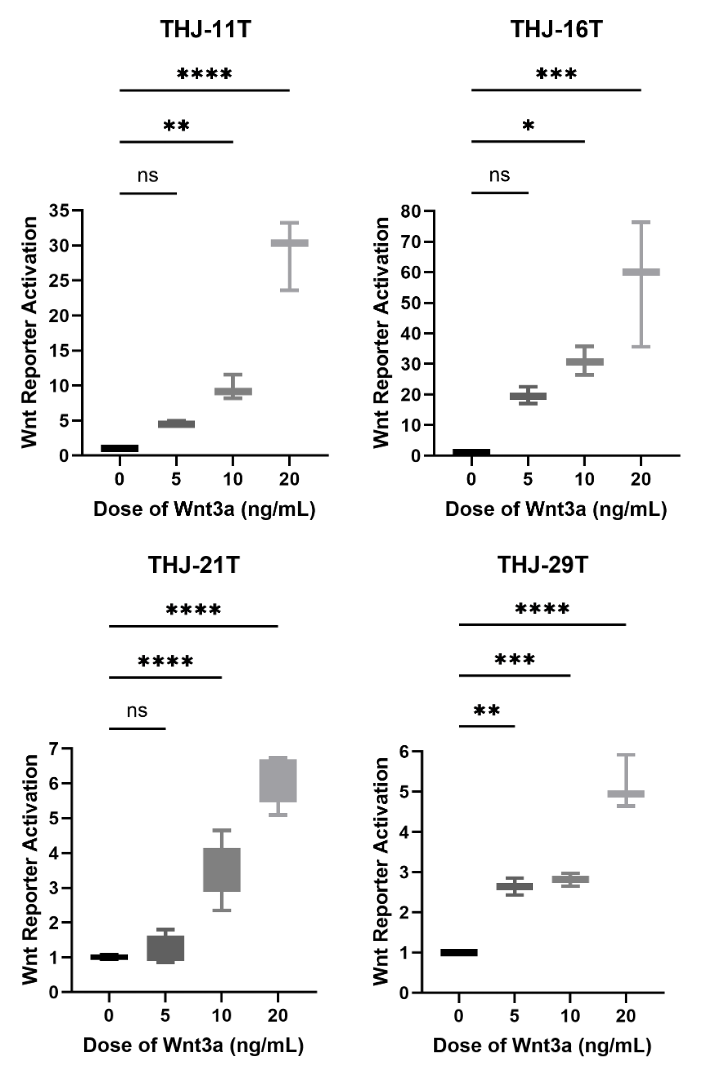


**Supplemental Table I**: Driver mutations of each cell line used in this manuscript.

| Cell Lines | Driver Mutations |
| --- | --- |
| THJ – 21T^1–5^ | BRAF^[[1]](#footnote-1)^ c.1799T>A, TERT^[[2]](#footnote-2)^ c.-124C>T, TP53^[[3]](#footnote-3)^ c.839G>C |
| THJ -16T^1–6^ | PIK3CA^[[4]](#footnote-4)^ c.1633G>A, TERT c.-124C>T, EP300^[[5]](#footnote-5)^ c.2396delC, RET^[[6]](#footnote-6)^ c.268G>A, TP53 c.818G>A, MKRN1::BRAF |
| THJ - 11T^2–4^ | KRAS^[[7]](#footnote-7)^ c.35G>T, TERT c.-124C>T |
| THJ – 29T^1-6^ | TERT c.-146C>T, TP53 c.310C>T, CDKN2A^[[8]](#footnote-8)^ c.207delG, HDAC10^[[9]](#footnote-9)^ c.399delG, FGFR2::OGDH |

**References**

1. Landa, I. *et al.* Comprehensive Genetic Characterization of Human Thyroid Cancer Cell Lines: A Validated Panel for Preclinical Studies. *Clin. Cancer Res.* **25**, 3141–3151 (2019).

2. Marlow, L. A. *et al.* Detailed Molecular Fingerprinting of Four New Anaplastic Thyroid Carcinoma Cell Lines and Their Use for Verification of RhoB as a Molecular Therapeutic Target. *J. Clin. Endocrinol. Metab.* **95**, 5338–5347 (2010).

3. Landa, I. *et al.* Frequent Somatic TERT Promoter Mutations in Thyroid Cancer: Higher Prevalence in Advanced Forms of the Disease. *J. Clin. Endocrinol. Metab.* **98**, E1562–E1566 (2013).

4. Li, W., Reeb, A. N., Sewell, W. A., Elhomsy, G. & Lin, R.-Y. Phenotypic Characterization of Metastatic Anaplastic Thyroid Cancer Stem Cells. *PLoS ONE* **8**, e65095 (2013).

5. Kasaian, K. *et al.* The genomic and transcriptomic landscape of anaplastic thyroid cancer: implications for therapy. *BMC Cancer* **15**, 984 (2015).

6. Von Roemeling, C. A. *et al.* Aberrant Lipid Metabolism in Anaplastic Thyroid Carcinoma Reveals Stearoyl CoA Desaturase 1 as a Novel Therapeutic Target. *J. Clin. Endocrinol. Metab.* **100**, E697–E709 (2015).

1. BRAF: NM_004333.6 [↑](#footnote-ref-1)
2. TERT: NM_198253.3 [↑](#footnote-ref-2)
3. TP53: NM_000546.6 [↑](#footnote-ref-3)
4. PIK3CA: NM_006218.4 [↑](#footnote-ref-4)
5. EP300: NM_001429.4 [↑](#footnote-ref-5)
6. RET: NM_020975.6 [↑](#footnote-ref-6)
7. KRAS: NM_004985.5 [↑](#footnote-ref-7)
8. CDKN2A: NM_000077.5 [↑](#footnote-ref-8)
9. HDAC10: NM_032019.6 [↑](#footnote-ref-9)
